# The Hypodermic Infection of Remedies

**Published:** 1868-12

**Authors:** 


### The Hypodermic Infection of Remedies.

In an excellent paper under the above heading (quoted from the *Practitioner* by the *California Medical Gazette*) Dr. Anstie gives the results of an extensive experience of the subcutaneous administration of medicines and lays down some valuable rules of practice. With regard to the danger which is supposed by some timorous persons to be involved in the use of the hypodermic syringe, the writer asserts that: "there is *absolutely* none if the injector will remember two cautions—first, that the physiological activity of nearly every substance which can be thus used is *three if not four times greater when it is given by the skin than when it is swallowed;* and secondly, that the liquid injected must not be either markedly acid, or markedly alkaline, nor in any way obviously *irritant to tissue.*" The advantages of the method are:—"1. Economy of the drug. 2. Entire abolition of the depressing or irritant effects which are locally produced in the alimentary canal during the *digestion* of various remedies. 3. Far greater *permanence of effect*, in many cases, than can be produced by medicine swallowed. 4. Much greater rapidity of action—a quality which makes injected remedies of priceless value in certain emergencies. One most important conclusion from these facts is this: *that anodynes and hypnotics ought never to be administered by the mouth in acute diseases attended with anarexia.* The practical importance of this principle is immense. Regular and systematic nutrition is the great necessity and the great difficulty in these diseases, and the avoidance of any treatment tending to interfere with digestion of simple food is a cardinal necessity.

After reviewing the discussion between Mr. Charles Hunter on the one part, and Behier and Eulenberg on the other, as to the local or general effects of hypodermic injections, Dr. Anstie is inclined to agree with the former (who holds that the locality selected for injection is a matter of indifference) with certain modifications in exceptional cases, his conclusion being that: "when the painful part is a convenient place for injection, it is as well to perform it locally; and that in rheumatic and other cases in which, from thickening of tissues round the nerve, the process of absorption is slower, it is desirable to do this even at considerable inconvenience, since the local effect is probably considerable; but that in the vast majority of cases it is absolutely indifferent, as regards the effect on the pain, where we inject, provided we select a favorable place for absorption, and that in these cases it will be desirable to vary the place of injection each time, in order to avoid local irritation and thickening."

The drugs from which Dr. Anstie has derived the most useful results are morphia, sulphate of atropine, veratine, caffeine, camphor, Indian hemp, and strychnine, as hypnotics or anodynes; quinine both as anodyne and antiperiodic; prussic acid as a calmative in vomiting; corrosive sublimate in congenital syphilis; arsenic and atropine as anti-spasmodics. Opium, codcia, aconitine, and podophylin, he has found either inert or superfluous. Woorara and nicotine have been successfully used by others for tetanus; conia for asthma and angina; digitalis for various febrile conditions; tartrate of antimony as an emetic, etc.

Morphia should be used in the form of a solution of the acetate, dissolved with a minimum of acetic acid in hot distilled water, five grains to the drachm. A stronger solution can be made by the use of glycerine, but the author suspects with Dr. Lawson, that glycerine diminishes the physiological activity of morphia salts. Of the five-grain solution, one minim, containing one-twelfth of a grain, is a useful minimum dose in cases of slight neuralgic pain. Two minims is the best commencing dose for the relief of severe pain, and as a hypnotic in states of nervous irritability. Three minims (or $\frac{1}{4}$ grain) is an *unsafe dose to commence with;* dangerous and even fatal results having been known to follow its use. It is rarely advisable to increase the dose above six minims ($\frac{1}{2}$ grain) except in the case of persons habituated to indulgence in opiates. In acute diseases, and especially in delirium tremens, subcutaneous injection of morphia should always be preferred to the administration of opiates by the mouth. A noticeable fact in connection with the hypodermic use of morphia is the far greater permanence of its action in neuralgic pain than when taken into the stomach. Equally remarkable is its apparent "antiphlogistic" effect in threatening pericarditis, pleurisy, etc.

A solution of sulphate of atropine, four minims containing one-sixtieth of a grain, is extremely useful for the relief of local pain and spasm. Two minims (or 1-120 of a grain) is the proper commencing dose for adults unless the pain to be relieved is very severe. It should be cautiously increased to 1-60 or 1-50 of a grain. The appearance of the slightest symptoms of atropism, such as dryness in the throat, and diplopia, (which have some times supervened upon doses as small as the 1-240 of a grain) should warn us of the danger of increasing the dose. In some cases persons who are unable to bear morphine will tolerate atropine, and *vice versa.* Dr. Anstie anticipates the most valuable results from the hypodermic use of atropine in painful irritis, and especially in threatening glaucoma. He thinks that the development of the latter affection was prevented in two cases under his care by injections of one-sixtieth of a grain. "Finally," he says, "I may remark of atropine that it is incomparably the best of all medicinal remedies for every kind of pain in the pelvic viscera. Nothing can approach it in this respect."

Strychnia is used by Dr. Anstie in cases very different from those in which exclusively it has been employed by others. He doubts its applicability to paralysis, but has found it of great value in certain varieties of neuralgic pain, such as gastralgia and neuralgia of the heart. The best form is a solution of the sulphates, two grains to the ounce of distilled water, and the proper commencing dose is two minims (1-120 of a grain.)

Caffeine, injected in doses of one grain is strongly recommended for neuralgia and for insomnia from chronic alcoholism.—*Medical Gazette.*

